# *DRD2* and *PPP1R1B* (*DARPP-32*) polymorphisms independently confer increased risk for autism spectrum disorders and additively predict affected status in male-only affected sib-pair families

**DOI:** 10.1186/1744-9081-8-19

**Published:** 2012-05-04

**Authors:** Joe A Hettinger, Xudong Liu, Melissa L Hudson, Alana Lee, Ira L Cohen, Ron C Michaelis, Charles E Schwartz, Suzanne ME Lewis, Jeanette JA Holden

**Affiliations:** 1 Department of Physiology, Queen’s University, Kingston, ON, Canada; 2 Queen’s Genetics and Genomics Lab at Ongwanada, Ongwanada Resource Centre, Kingston, ON, Canada; 3 Department of Psychiatry, Queen’s University, Kingston, ON, Canada; 4 Autism Spectrum Disorders – Canadian-American Research Consortium, Kingston, ON, Canada; 5 Department of Psychology and George A. Jervis Clinic, New York State Institute for Basic Research in Developmental Disabilities, Staten Island, NY, USA; 6 Department of Biology, Western Carolina University, Cullowhee, North Carolina, USA; 7 Center for Molecular Studies, Greenwood Genetic Center, Greenwood, South Carolina, USA; 8 Department of Medical Genetics, University of British Columbia, Vancouver, BC, Canada; 9 B.C. Child and Family Research Institute, Vancouver, BC, Canada; 10 Centre for Neuroscience Studies, Queen’s University, Kingston, ON, Canada; 11 Autism Research Program/Genetics and Genomics Research Laboratory, Ongwanada Resource Centre, 191 Portsmouth Ave, Kingston, ON, Canada, K7M 8A6

**Keywords:** Autism spectrum disorders, Dopamine receptors, DARPP-32, Association study, Candidate gene

## Abstract

**Background:**

The neurotransmitter dopamine (DA) modulates executive functions, learning, and emotional processing, all of which are impaired in individuals with autism spectrum disorders (ASDs). Our previous findings suggest a role for dopamine-related genes in families with only affected males.

**Methods:**

We examined two additional genes which affect DA function, the *DRD2* and *PPP1R1B* (*DARPP-32*) genes, in a cohort of 112 male-only affected sib-pair families. Selected polymorphisms spanning these genes were genotyped and both family-based and population-based tests were carried out for association analysis. General discriminant analysis was used to examine the gene-gene interactions in predicting autism susceptibility.

**Results:**

There was a significantly increased frequency of the *DRD2* rs1800498TT genotype (*P* = 0.007) in affected males compared to the comparison group, apparently due to over-transmission of the T allele (*P* = 0.0003). The frequency of the *PPP1R1B* rs1495099CC genotype in affected males was also higher than that in the comparison group (*P* = 0.002) due to preferential transmission of the C allele from parents to affected children (*P* = 0.0009). Alleles rs1800498T and rs1495099C were associated with more severe problems in social interaction (*P* = 0.0002 and *P* = 0.0016, respectively) and communication (*P* = 0.0004 and *P* = 0.0046), and increased stereotypic behaviours (*P* = 0.0021 and *P* = 0.00072). General discriminant analysis found that the *DRD2* and *PPP1R1B* genes additively predicted ASDs (*P* = 0.00011; Canonical R = 0.26) and explain ~7% of the variance in our families. All findings remained significant following corrections for multiple testing.

**Conclusion:**

Our findings support a role for the *DRD2* and *PPP1R1B* genes in conferring risk for autism in families with only affected males and show an additive effect of these genes towards prediction of affected status in our families.

## Introduction

Autism spectrum disorders (ASDs) are characterized by repetitive behaviours and interests, as well as deficiencies in communication and social interaction. They are believed to be complex, polygenic disorders predominantly characterized by multifactorial inheritance [1], although Zhao et al. [2] (2007) suggested that Mendelian inheritance may apply to autism risk in a subgroup of families with affected males. To address the significant genetic heterogeneity and phenotypic variation seen among affected individuals, which has confounded the conclusive identification of candidate genes for the majority of cases, we have been testing genes for evidence of association with specific ASD endophenotypes in an effort to identify a subgroup within the ASD population whose members share an underlying pathophysiology.

Abnormalities in neurotransmitter pathways can account for the deficits seen in persons with ASDs. In contrast to the attention which has been directed to the study of genes involved in the glutamate [3], GABA [3] and serotonin pathways [1], genes related to the synthesis, function and metabolism of dopamine (DA) have received little attention [4].

We have argued [5] that genes in the dopaminergic (DAergic) pathway are excellent candidates based on their affect on ASD behaviours. DA modulates motor functions [6], cognitive processes (including executive functions [7] and learning [8]), and emotional regulation [9] - all of which are abnormal in individuals with autism [10-13]. DA also plays a role in social interactions [14] and the pathophysiology of stereotypies [15]; impairments in social interaction and the presence of increased stereotypies are core features of autism. Furthermore, there is decreased DAergic activity in the medial prefrontal cortex (PFC) in children with autism [16], and increased levels of the major metabolite of DA, homovanillic acid (HVA), in cerebrospinal fluid from affected children compared to controls [17], indicating altered DAergic function in these individuals.

Based on our earlier findings on the dopamine β-hydroxylase (*DBH*) gene, which encodes the enzyme that converts DA to norepinephrine, in mothers from male-only affected sib-pair families [18], we have pursued a comprehensive study of DA-related genes in mothers and sons with ASDs. Since our initial study with *DBH,* in which we found an increased frequency of the 19-bp deletion in mothers from male-only affected sib-pair families [18], we identified a 3-marker risk haplotype in the dopamine D1 receptor (*DRD1*) gene [5] in our family cohort having only affected sons. Here we report our findings on two other genes affecting DA levels and function, the dopamine D2 receptor (*DRD2*) and protein phosphatase 1, regulatory subunit 1B (*PPP1R1B*) genes, and results of tests for gene-gene interactions.

The *DRD2* gene comprises eight exons [19] and maps to 11q22-q23 [20]. It encodes the dopamine D2 receptor which, in addition to its role in postsynaptic neurons, acts as an autoreceptor mediating DA synthesis [21] and neurotransmission [22] in DAergic neurons. The dopamine D2 receptor is involved in the DAergic modulation of executive functions [23], reversal learning [24] and emotional processing [25]. *Drd2*−/− mice have abnormal gait similar to that of individuals with Parkinson disease [26], and the administration of antipsychotic medications (e.g., risperidone, a dopamine D2 receptor antagonist) has proven efficacious in treating symptoms associated with ASDs [27].

The *PPP1R1B* gene, located at chromosome 17q12 and comprising 7 exons ( http://www.ncbi.nlm.nih.gov; GeneID 84152), encodes DARPP-32, which is expressed in dopaminoceptive (DAceptive) neurons [28] and mediates the effects of both D1 and D2 dopamine receptor classes [29]. For example, dopamine D2 receptor antagonist-induced catalepsy in rats is attenuated in *Ppp1r1b*−/− mice [30] and knockout mice are impaired in reversal learning [31]. Genetic [32,33] and immunoblot [34] studies showed an association of *PPP1R1B* with altered PFC DARPP-32 protein levels in schizophrenia and bipolar disorder, two conditions for which DA dysfunction is evident and which exhibit comorbidity with autism [35].

There are two previous studies which examined the *DRD2* gene as a candidate gene for autism. The first [36] reported an increased frequency of the TaqI A1 allele in persons with autism (N = 33) compared to controls (N = 314), whereas the second [37] found no evidence for transmission disequilibrium of an intragenic microsatellite in 39 affected sib-pair families. No association studies have examined the role of *PPP1R1B* as a candidate gene for ASDs.

Based on our hypothesis that DA-related genes are important in male-only affected sib-pair families [18], we examined four markers at the *DRD2* locus that are commonly used to investigate possible associations between DAergic function and behavioural abnormalities [38], and three polymorphisms at the *PPP1R1B* locus to determine whether there was an association of these DA-related genes with autism.

## Materials and methods

### Subjects

The 112 affected sib-pair families and the comparison group (N = 443) were previously described [5]. Briefly, the study group included 28 families from Canada [18], 5 from the South Carolina Autism Project [39], and 79 families obtained through the Autism Genetic Resource Exchange (AGRE) in the United States [40]. All families have two or more children with either autism or an ASD, including Asperger syndrome and pervasive developmental disorder (PDD) variants. This study was approved by the Queen’s University Research Ethics Board and written informed consent was obtained from parents of all participating families from Canada and South Carolina, and through AGRE [40].

All 443 samples from the comparison group (bloodspots from anonymous newborns taken for the purpose of PKU testing and made available from the Ontario Ministry of Health) were available for the study of the *PPP1R1B* locus. Two hundred and fifty-three comparison group samples were used for the *DRD2* gene studies. There were no significant differences in the allele frequencies for *DRD2* (*P* = 0.57–0.93) and *PPP1R1B* (*P* = 0.28–0.91) markers in males and females from the respective comparison cohorts and thus our comparison cohorts included both males and females. Although comprehensive information regarding psychiatric and behavioural disorders is not available for the comparison group, we do not expect the prevalence of ASDs in this comparison group to be greater than that in the general population, or approximately 1/110 [41].

### Marker amplification and genotyping

#### DRD2

**Figure 1:**
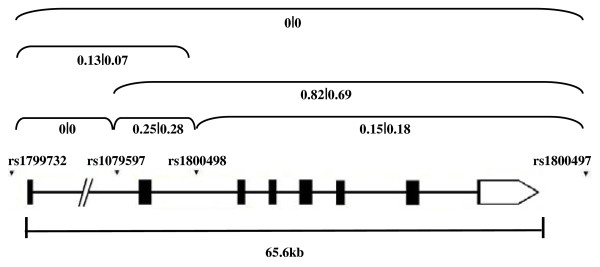
**Illustration of the *DRD2 *locus. **A schematic showing gene structure, marker positions and measures of linkage disequilibrium between rs1799732, rs1079597, rs1800498 and rs1800497 listing r^2^ of the comparison group (N=244) followed by r^2^ of parents from families (N=213). Legend: ▪ exon; ━ intron; □ untranslated region.

Four polymorphisms (rs1799732 Ins/Del, rs1079597 G/A, rs1800498 T/C and rs1800497 C/T) were studied at the *DRD2* locus (Figure 1). The *DRD2* gene contains four haplotype blocks [Haploview 4.1; available at http://hapmap.ncbi.nlm.nih.gov] [42]. Three of the blocks are small (<4 kb) and one block is 20 kb in size. One of four polymorphisms used in this study, rs1079597, is part of the HapMap dataset. both this SNP and and rs1800498 are located within the larger haplotype block and were examined in previous studies of other neuropsychiatric disorders. Primer sequences, PCR and digestion conditions are shown in Table 1. All PCR reactions were carried out using 5 ng of template DNA; digestion products were separated on either 2% (rs1079597, rs1800498 and rs1800497) or 2.5% (rs1799732) agarose gels and visualized using ethidium bromide and UV illumination. In order to minimize genotyping errors, DNAs from affected individuals and family members were randomly arranged on 96-well plates, and all results were independently scored and tabulated by two persons.

**Table 1:** **PCR and digestion conditions for *DRD2 *and *PPP1R1B *markers used in this study**

***DRD2* Marker (5′ → 3′)**^**1**^	**Primers**	**[MgCl**^**2**^**]**	**Annealing temperature**	**# of cycles**	**Restriction enzyme (U)**^**2**^
rs1799732 Ins/Del	F 5′-GAGAAGACTGGCGAGCAGAC-3′	1.5 mM	63°C	35	BstNI (0.05)
	R 5′-CCACCAAAGGAGCTGTACCT-3′				
rs1079597 G/A^3^	F 5′-GATACCCACTTCAGGAAGTC-3′	1.0 mM	55°C	34	TaqI (0.4)
	R 5′-CAGTAAAGAACTAGGAGTCAG-3′				
rs1800498 T/C^3^	F 5′-CCCAGCAGGGAGAGGGAGTA-3′	1.0 mM	55°C	34	TaqI (0.4)
	R 5′-GACAAGTACTTGGTAAGCATG-3′				
rs1800497 C/T^3^	F 5′-CCGTCGACGGCTGGCCAAGTTGTCTA-3′	1.0 mM	58°C	34	TaqI (0.4)
	R 5′-CCGTCGACCCTTCCTGAGTGTCATCA-3′				
***PPP1R1B *Marker (5′ → 3′)**	**Primers**	**[MgCl**^**2**^**]**	**Annealing temperature**	**# of cycles**	**Restriction enzyme (U)**^**2**^
rs1495099 G/C	F 5′-TTGTTGCTGAGCTGAGATGC-3′	1.0 mM	60°C	35	PvuII (0.3)
	R 5′-CTCCAGGGAAATGCACAAAG-3′				
rs907094 T/C	F 5′-ACCTGATTGGGAGAGGGACT-3′	1.0 mM	60°C	34	MseI (0.3)
	R 5′-GTAAGCTGAGGGGCCTGTG-3′				
rs3764352 A/G	F 5′-CTGTTTTGGAGGGGTCTCAG-3′	1.0 mM	60°C	35	BccI (0.3)
	R 5′-TGGGAATACTGAAGAGTCAACC-3′				

^1^Rs1799732 Ins/Del, rs1079597 G/A, rs1800498 T/C, and rs1800497 C/T previously known as −141 C Ins/Del, TaqI B, TaqI D, and TaqI A, respectively.

^2^Restriction enzymes obtained from New England Biolabs, Pickering, ON, Canada

^3^Primer sequences have been reported previously [43].

#### PPP1R1B

**Figure 2:**
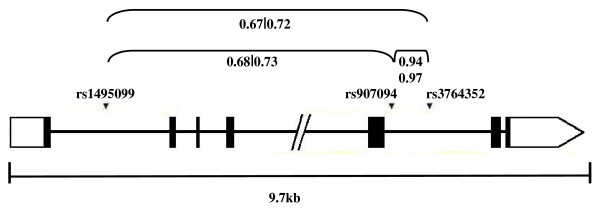
**Illustration of the *PPP1R1B *locus. **A schematic showing gene structure, marker positions and measures of linkage disequilibrium between rs1495099, rs907094 and rs3764352 listing r^2^ of the comparison group (N=435) followed by r^2^ of parents from families (N=216). Legend: ▪ exon; ━ intron; □ untranslated region.

Three polymorphisms (rs1495099 G/C, rs907094 T/C and rs3764352 A/G) were examined in the *PPP1R1B* gene (Figure 2). These variants were chosen from the NCBI dbSNP Build 121 database from the Human Genome Project (available at http://www.ncbi.nlm.nih.gov/SNP/snp_summary.cgi) based on the following criteria: the markers span the *PPP1R1B* locus, they have minor allele frequencies (MAFs) of approximately 20%, and alleles at rs907094 and rs3764352 are associated with changes in DARPP-32 mRNA expression and measures of cognitive performance [32]. The *PPP1R1B* locus has a single haplotype block which includes rs907094 and rs3764352 as haplotype-tagged SNPs (htSNPs). PCR reactions were carried out using 5 ng of template DNA and amplicons were digested using conditions shown in Table 1. All digestion products were separated on 2% agarose gels and visualized using ethidium bromide and UV illumination. In order to minimize genotyping errors, DNAs from affected individuals and family members were randomly arranged on 96-well plates, and all results were independently scored and tabulated by two persons.

### Statistical analyses

Prior to carrying out analyses, Mendelian errors were checked in the family cohort using the FBAT program, v1.5.5 [44]. *DRD2* marker data on five families and *PPP1R1B* marker data on two families were excluded from the analyses due to identified Mendelian errors. Single gene analyses were performed as previously described [5]. To avoid allele and genotype frequency distortion from using related individuals in case–control comparisons, one affected individual was selected at random from each family using SPSS v14.0 (SPSS, Chicago, IL) with the same cohort of randomly chosen individuals used for single marker allele and genotype frequency comparisons for all polymorphisms as well as general discriminant analyses. Family-based association tests (FBAT), including quantitative disequilibrium tests (QTDT), were done using FBAT v1.5.5. Because FBAT v1.5.5 can accommodate multiple affected individuals from each family, all affected individuals including those used for case–control comparisons, were included for FBAT and QTDT analyses. The domains, ‘reciprocal social interaction’, ‘communication’ and ‘repetitive stereotyped behaviours’ used for QTDT analyses were derived from the total scores from the ‘Qualitative Abnormalities in Reciprocal Social Interaction’ (A1 to A4), ‘Qualitative Abnormalities in Communication’ (B1, B2(V), B3(V) and B4) and ‘Restricted, Repetitive, and Stereotyped Patterns of Behaviour’ (C1 to C4) subdomains in the ADI-R diagnostic algorithm [45].

General Discriminant Analysis (GDA) was used to evaluate the predictive value of our single gene findings in discriminating between individuals with and without autism, as well as to test for evidence of interaction effects between genes. Genotypes were coded as categorical variables and GDA was performed using Statistica 9.1 [Statsoft, Tulsa, OK, USA].

### Corrections for multiple comparisons

The contribution of a single gene to autism susceptibility is predicted to be relatively small and thus difficult to detect statistically. Although corrections for multiple testing must be made in genetic association studies, Bonferroni correction is thought to be too stringent, with a high risk for rejecting true significant findings. The false discovery rate (FDR) approach [46] is a compromise between not correcting for multiple comparisons, which is too lax, and Bonferroni adjustments, which are too strict. FDR has two methods, the Benjamini and Hochberg (BH) method and the Benjamini and Liu method (BL) [46]. The BH method is appropriate for correcting for both independent and positively-dependent comparisons [47], and thus is appropriate for genetic studies using polymorphisms.

FDR corrections were performed separately for single gene case–control and family-based comparisons, as well as GDA findings, using an initial FDR threshold of 0.050.

## Results

### Linkage disequilibrium of polymorphisms at the *DRD2* and *PPP1R1B* loci

High r^2^ measures of linkage disequilibrium (LD) were observed between *DRD2* polymorphisms rs1079597 and rs1800497, with no LD between rs1799732 and rs1079597 and rs1800497 (Figure 1). R-squared measures between *PPP1R1B* markers showed high LD (r^2^ > 0.9) between rs907094 and rs3764352, and lower LD between rs1495099 and rs907094 and rs3764352 (Figure 2).

### Case–control comparisons

All four markers of *DRD2* were in HWE in the comparison and family cohorts with the exception of rs1799732 and rs1800498 in affected males (*P* = 0.009 and *P* = 0.012, respectively); all markers were in HWE in the parents from these families. As shown in Table 2, there was an increased frequency of the rs1800498 TT genotype (*P* = 0.007) in affected males (43.4% versus 28.7% in the comparison group), which remained significant following FDR correction. No significant differences in genotype frequencies of the other three markers were seen between cases and the comparison group (*P* = 0.32–0.51) (Table 2). Separate analyses using the extended-transmission disequilibrium test (ETDT) showed that significant over-transmission of the rs1800498 T allele was not from mothers (21 transmitted, 11 untransmitted; *χ*^2^ = 3.125, df = 1, *P* = 0.077) but was from fathers (26 transmitted, 12 untransmitted; *χ*^2^ = 5.158, df = 1, *P* = 0.023) to affected sons. No differences in rs1800498 T allele or rs1800498 TT genotype frequencies were found between mothers (*P* = 0.69 and *P* = 0.26, respectively) or fathers (*P* = 0.90 and *P* = 0.65, respectively) and the comparison group (data not shown).

**Table 2:** **Marker genotype frequencies at the *DRD2 *and *PPP1R1B *loci in the comparison group and males with ASD from affected sib-pair families**^1^

***DRD2***			**Genotype**				**FDR threshold**^**2**^
rs1799732	N	Ins/Ins	Del/Ins	Del/Del	*χ*^2^ (df = 2)	*P*^1^	
Comparison group	238	188 (79.0%)	46 (19.3%)	4 (1.7%)			
Affected males^3^	109	89 (81.7%)	16 (14.7%)	4 (3.7%)	2.253	0.32	0.025
rs1079597	N	G/G	A/G	A/A	*χ*^2^ (df = 2)	*P*	
Comparison group	244	168 (68.9%)	71 (29.1%)	5 (2.0%)			
Affected males^3^	105	70 (66.7%)	30 (28.6%)	5 (4.8%)	1.944	0.38	0.038
rs1800498	N	T/T	C/T	C/C	*χ*^2^ (df = 2)	*P*	
Comparison group	244	70 (28.7%)	130 (53.3%)	44 (18.0%)			
Affected males^3^	106	46 (43.4%)	38 (35.8%)	22 (20.8%)	9.790	**0.007**	0.013
rs1800497	N	C/C	T/C	T/T	*χ*^2^ (df = 2)	*P*	
Comparison group	245	164 (66.9%)	69 (28.2%)	12 (4.9%)			
Affected males^3^	107	65 (60.7%)	35 (32.7%)	7 (6.5%)	1.333	0.51	0.050
***PPP1R1B***		**Genotype**					**FDR threshold**^**2**^
rs1495099	N	C/C	C/G	G/G	*χ*^2^ (df = 2)	*P*^1^	
Comparison group	434	43 (9.9%)	163 (37.6%)	228 (52.5%)			
Affected males^3^	109	24 (22.0%)	39 (35.8%)	46 (42.2%)	12.273	**0.002**	0.0083
rs907094	N	C/C	C/T	T/T	*χ*^2^ (df = 2)	*P*	
Comparison group	434	30 (6.9%)	146 (33.6%)	258 (59.4%)			
Affected males^3^	110	16 (14.5%)	38 (34.5%)	56 (50.9%)	7.176	**0.028**	0.033
rs3764352	N	G/G	A/G	A/A	*χ*^2^ (df = 2)	*P*	
Comparison group	434	29 (6.7%)	152 (35.0%)	253 (58.3%)			
Affected males^3^	110	16 (14.5%)	38 (34.5%)	56 (50.9%)	7.408	**0.025**	0.025

^1^P-values less than 0.05 are in bold and P-values which remain significant following false-discovery rate (FDR) corrections for multiple comparisons are underlined

^2^P-value ≤ FDR threshold is significant

^3^One affected individual was randomly chosen from each family

All three *PPP1R1B* markers were in HWE in the comparison group; none were in HWE in the cohort of affected individuals (*P* = 0.008, *P* = 0.033 and *P* = 0.033, respectively), although all markers were in HWE in the parents (data not shown). As shown in Table 2, the rs1495099 CC (*P* = 0.002), rs907094 CC (*P* = 0.028) and rs3764352 GG (*P* = 0.025) genotype frequencies were increased in affected males (22.0%, 14.5% and 14.5%, respectively) relative to the comparison group (9.9%, 6.9% and 6.7%, respectively). Findings on alleles of these polymorphisms were similar, with the minor allele frequencies of all three markers, rs1495099 C, rs907094 C and rs3764352 G, being increased in the affected males relative to the comparison group (*P* = 0.001, *P* = 0.014 and *P* = 0.021, respectively, all remained significant following FDR correction; data not shown).

Because we hypothesize that maternal effects including genetic factors may contribute to autism susceptibility in some autism families [18], we compared frequencies of rs1495099 C alleles and rs1495099 CC genotypes between mothers (33.2% and 12.7%, respectively) and the comparison group (28.7% and 9.9%, respectively) but found no significant differences (*P* = 0.19 and *P* = 0.39, respectively; data not shown).

### Family-based association tests

FBAT showed that the DRD2 rs1800498 T allele was over-transmitted to affected males (*P* = 0.0003; significant following FDR correction), while no evidence of preferential allele transmission was found for the other three markers (*P* = 0.16–0.94) (Table 3). The rs1799732 Ins - rs1079597 G - rs1800498 T - rs1800497 C (Ins-G-T-C) haplotype, consisting of the major alleles for all four markers, was over-transmitted to affected males but with a P-value (*P* = 0.0009; data not shown) slightly higher than that observed with rs1800498 T alone (*P* = 0.0003). It should be noted that the additive model was used in FBAT for polymorphisms at the *DRD2* locus because of the increased CT and TT genotype frequencies found for rs1800498.

**Table 3:** **FBAT of marker allele transmissions under an additive model at the *DRD2 *locus and FBAT of marker allele transmissions under a recessive model at the *PPP1R1B *locus in affected sib-pair families**^**1**^

***DRD2***	**# Fam**	**Observed**	**Expected**	**Z**	***P*^**2**^**	**FDR threshold**^**3**^
rs1799732						
Ins	31	90.0	84.0	1.4	0.16	0.030
Del	31	38.0	44.0	−1.4	0.16	
rs1079597						
G	56	149.0	148.0	0.17	0.86	0.040
A	56	71.0	72.0	−0.17	0.86	
rs1800498						
T	73	185.0	160.0	3.6	**0.0003**	0.010
C	73	115.0	140.0	−3.6	**0.0003**	
rs1800497						
C	63	171.0	170.5	0.08	0.94	0.050
T	63	89.0	89.5	−0.08	0.94	
***PPP1R1B***	**# Fam**	**Observed**	**Expected**	**Z**	***P***^**2**^	**FDR threshold**^**3**^
rs1495099						
G	52	42.0	43.8	−0.4	0.72	0.043
C	34	39.0	26.3	3.3	**0.00092**	0.0071
rs907094						
T	52	46.0	48.3	−0.5	0.66	0.036
C	24	25.0	19.8	1.6	0.11	0.029
rs3764352						
A	54	48.0	49.8	−0.3	0.73	0.050
G	26	27.0	21.3	1.7	0.09	0.021

^1^All affected individuals were included in the analyses.

^2^P-values less than 0.05 are in bold and P-values which remain significant following false-discovery rate (FDR) corrections for multiple comparisons are underlined.

^3^P-value ≤ FDR threshold is significant.

For *PPP1R1B*, a recessive model was used in FBAT because of the increased frequency of rs1495099 CC, rs907094 CC and rs3764352 GG genotypes found in affected individuals compared to the comparison cohort. Family-based association analyses and FDR-based corrections showed significant over-transmission of rs1495099 C (*P* = 0.00092) but not of rs907094 C (*P* = 0.11) or rs3764352 G (*P* = 0.09) (Table 3). The rs1495099 C - rs907094 C - rs3764352 G (C-C-G) haplotype was not significantly over-transmitted to affected males (*P* = 0.031; not significant following FDR correction; data not shown) compared to that of rs1495099 C alone (*P* = 0.00092).

### Genotype-phenotype associations

We next used quantitative transmission disequilibrium tests (QTDT) to determine whether the extent of impairment in the core behaviours was more pronounced in affected males with the risk allele. The *DRD2* rs1800498 T allele was associated with more severe impairments in reciprocal social interaction (*P* = 0.0002), verbal communication (*P* = 0.0004), and repetitive and stereotyped behaviours (*P* = 0.0021); these findings remained significant following corrections for multiple comparisons.

The *PPP1R1B* rs1495099 C allele was associated with higher ADI-R domain scores (more severe problems) in affected males for social interaction (*P* = 0.0016), nonverbal communication (*P* = 0.0046), and stereotyped behaviours (*P* = 0.00072) (Table 4), with strong evidence for association shown by multivariate QTDT between rs1495099 C and the combined effect of all three ADI-R subdomains (*P* = 0.00042; data not shown). All findings were significant following FDR-based corrections.

**Table 4:** **QTDT of rs1800498 alleles under an additive model at the *DRD2 *locus and QTDT of rs1495099 alleles under a recessive model at the *PPP1R1B *locus in affected sib-pair families**^**1**^

**ADI-R Subdomain**	***DRD2 *rs1800498**	**# Fam**	**Observed**	**Expected**	**Z**	***P***^**2**^	**FDR threshold**^**3**^
Social	T	56	2909.0	2452.5	3.7	**0.0002**	0.017
Interaction	C	56	1557.0	2013.5	−3.7	**0.0002**	
Verbal	T	46	1364.0	1103.5	3.6	**0.0004**	0.033
Communication	C	46	666.0	926.5	−3.6	**0.0004**	
Stereotyped	T	56	876.0	754.5	3.1	**0.0021**	0.050
Behaviours	C	56	502.0	623.5	−3.1	**0.0021**	
**ADI-R Subdomain**	***PPP1R1B rs1495099***	**# Fam**	**Observed**	**Expected**	**Z**	***P***^**2**^	**FDR threshold**^**3**^
Social	C	19	480.0	283.5	3.2	**0.0016**	0.017
Interaction	G	36	535.0	579.5	−0.5	0.59	0.042
Nonverbal	C	10	108.0	52.3	2.8	**0.0046**	0.025
Communication	G	20	130.0	123.3	0.2	0.82	0.050
Stereotyped	C	19	142.0	79.8	3.4	**0.00072**	0.0083
Behaviours	G	36	176.0	200.3	−0.9	0.38	0.033

^1^All affected males were included for QTDT analyses

^2^P-values less than 0.05 are in bold and P-values which remain significant following false-discovery rate (FDR) corrections for multiple comparisons are underlined

^3^P-value ≤ FDR threshold is significant

### General discriminant analyses

We used GDA to determine whether DA-related genes predict ASD susceptibility in affected males compared to the comparison group and to test for gene-gene interactions. Tests were performed based on our single gene findings for *DRD2* and *PPP1R1B* from this study, and our previous findings with *DRD1*[5]. *DRD2* rs1800498 genotypes and *PPP1R1B* rs1495099 genotypes each significantly contributed to prediction of ASDs in our families (*P* = 0.0063 and *P* = 0.00086, respectively), as well as when weighted and tested together (*P* = 0.00011; Canonical R = 0.26) (Table 5). We generated a classification matrix to determine the percent correct classification of individuals with and without autism based on *DRD2* rs1800498 and *PPP1R1B* rs1495099 genotypes, and found that 97% of individuals from our comparison cohort and 13% of individuals with autism were correctly classified (the analysis was based on the a priori baseline frequency of 70% of individuals without autism and 30% of individuals with autism). Using the weighted scores to predict group membership, we found that 72% of control individuals and 64% of affected individuals were predicted correctly using *DRD2* and *PPP1R1B* genotypes. However, while ~7% of the variance was explained with *DRD2* rs1800498 and *PPP1R1B* rs1495099 genotypes, addition of our previously identified *DRD1* rs265981–rs4532–rs686 haplotypes to the analyses did not result in a significant improvement in the overall canonical correlation (Canonical R = 0.27).

**Table 5:** **General discriminant analysis of *DRD2 *rs1800498 and *PPP1R1B *rs1495099 genotypes towards prediction of ASDs in affected males**^**1**^

	**Eigenvalue**	**Canonical R**	**Wilk’s Lambda**	***χ***^**2**^** F**^**2**^	**df**	***P***^**3**^	**FDR threshold**^**4**^
*DRD2* - *PPP1R1B*	0.07	0.26	0.94	23.27	4	**0.00011**	0.017
*DRD2*			0.97	5.14	343	**0.0063**	0.050
*PPP1R1B*			0.96	7.20	343	**0.00086**	0.033

^1^One affected individual was randomly chosen from each family

^2^Chi-statistic reported for additive test of *DRD2* and *PPP1R1B* and F statistic reported for single gene tests

^3^P-values less than 0.05 are in bold and P-values which remain significant following false-discovery rate (FDR) corrections for multiple comparisons are underlined.

^4^P-value ≤ FDR threshold is significant

Adding all possible two-way interactions between *DRD2* rs1800498 and *PPP1R1B* rs1495099 genotypes, as well as comparisons between *DRD1* rs265981–rs4532–rs686 haplotypes and *DRD2* rs1800498 genotypes, and *DRD1* rs265981–rs4532–rs686 haplotypes and *PPP1R1B* rs1495099 genotypes, we found no evidence for gene-gene interactions (*P* = 0.35–0.75; data not shown).

## Discussion

Our model for the involvement of the DA pathway in determining some of the core deficits of ASDs is based on earlier results implicating the *DBH* gene as a maternal effect locus, and on our hypothesis that autism susceptibility is determined by a combination of fetal susceptibility genes and fetal gender as well as maternal effects including maternal genetic factors [18]. Following our findings with the *DRD1* gene [5], and as part of our investigation to determine whether other DA-related genes are significant factors in the etiology of ASDs, we found evidence for association of the *DRD2* and *PPP1R1B* genes with autism in affected males from multiple-incidence families.

We found an increased frequency of the *DRD2* rs1800498 TT genotype (*P* = 0.007) in affected males (43.4%) compared to the comparison group (28.7%) (Table 2), and the rs1800498 T allele was over-transmitted to affected children (*P* = 0.0003) (Table 3). The rs1800498 risk allele was associated with more severe impairments in social interaction (*P* = 0.0002), verbal communication (*P* = 0.0004) and stereotyped behaviours (*P* = 0.0021) in affected males (Table 4), and the rs1800498 TT genotype was associated with an increased risk for ASD with an OR of 1.9 [95% CI: 1.5–2.5]. In addition, we examined three polymorphisms at the *PPP1R1B* locus and identified the rs1495099 C allele as a recessive risk allele for susceptibility to ASDs in male-only affected sib-pair families. The CC genotype frequency was increased in affected males (22.0%) relative to the comparison group (9.9%, *P* = 0.002), and family-based association tests using FBAT with a recessive model showed distorted allele transmission with over-transmission of this allele in families (*P* = 0.00092) (Table 3). This allele was associated with greater impairments in social interaction (*P* = 0.0016) and nonverbal communication (*P* = 0.0046), and more severe stereotyped behaviours (*P* = 0.00072), core features of ASDs. Finally, the rs1495099 CC genotype was associated with an increased risk for ASD with an OR of 2.6 [95% CI = 1.9–3.6]. All findings were significant following FDR-based corrections for multiple comparisons.

### Functional effects of *DRD2* and *PPP1R1B* risk alleles on gene expression

Our findings at the *DRD2* and *PPP1R1B* loci may reflect the functional effects of unidentified risk variants in LD with rs1800498 at *DRD2* and rs1495099 at *PPP1R1B*. Functional analyses of these markers have not been reported but *in silico* analyses performed using PupaSuite [available at http://pupasuite.bioinfo.cipf.es/] [48] did not identify any putative functional role for these polymorphisms while analyses using FASTSNP [available at http:// http://fastsnp.ibms.sinica.edu.tw/pages/input_CandidateGeneSearch.jsp] [49] predicted a ‘very low-to-low’ effect for rs1800498 at *DRD2* as an intronic enhancer and a ‘very low-to-medium’ effect for rs1495099 at *PPP1R1B* as a regulatory region/intronic enhancer. Meyer-Lindenberg et al. [32] (2007) identified a common 7-marker *PPP1R1B* haplotype that was associated with increased DARPP-32 mRNA expression and improved performance on measures of working memory and cognitive flexibility. This haplotype included the T and A alleles of rs907094 and rs3764352 respectively, while a haplotype containing the minor alleles at these loci (i.e. rs907094 C and rs3754352 G) was associated with decreased mRNA expression in post-mortem brain. Houlihan et al. [50] (2009) screened this 7-marker haplotype to test *PPP1R1B* as a genetic determinant of cognitive ageing and found that rs907094 C and rs3754352 G alleles were associated with decreased cognitive ability. However, our findings do not support an association of either rs907094 C or rs3764352 G with autism in our family cohort. Unfortunately, because rs1495099 was not included in these studies, its functional role is not known. With respect to the *DRD2* locus, the rs1800498 polymorphism was found to be in low LD with rs1799732, a functional variant in the *DRD2* promoter [51], in our comparison group and parents from families (Figure 1). To investigate whether alleles from rs1799732 are contributing as a risk factor for autism susceptibility in our families, comparisons between family-based tests of rs1800498 and rs1799732 alleles considered separately (*P* = 0.0003 and *P* = 0.16, respectively), and haplotypes containing alleles from both rs1800498 and rs1799732, showed that the observed over-transmission in families is derived from rs1800498, and not because of the rs1799732 polymorphism (data not shown). However, only 31 families were informative for rs1799732 compared to 73 families for rs1800498, so we cannot determine from these findings whether alleles from the functional variant rs1799732 are contributing to autism susceptibility in our families. Another genetic variant at the *DRD2* locus, rs1076560GT, has been associated with altered mRNA isoform expression [52,53] and differences in striatal post-synaptic D2 receptor abundance [54]. Bertolini et al. [53] (2009) found in control and schizophrenia cohorts (N = 114 and N = 91, respectively) that individuals heterozygous for the minor “T” allele perform worse in the N-back test of working memory but only at a high level of difficulty (2-back) compared to individuals homozygous for the major “G” allele while Zhang et al. [52] (2007) found using healthy subjects (N = 117) that heterozygous individuals performed worse at higher attentional loads in the variable attentional control (VAC) task, and had increased activity as measured using BOLD fMRI in PFC and striatum compared to individuals homozygous for the major allele. However, the true contribution of this variant to DAergic function and cognition is unclear as no genotype effects of this polymorphism to overall working memory performance and fMRI activity were also reported [52,54]. Nevertheless, both rs1076560 and rs1079597 are in high LD in the HapMap CEU panel (r^2^ > 0.9) and are found in the same 20 kb haplotype block as rs1800498. However, no information is available regarding LD between rs1076560 and rs1800498. It is of interest that low LD (r^2^ < 0.3) was found between rs1800498 and rs1079597 in our comparison group and parents from families (Figure 1). Additional families informative at these *DRD2* loci are needed to determine whether these functional variants or another functional polymorphism in LD with rs1800498 is responsible for the increased risk for autism. In addition, functional analyses and resequencing of both genes in affected individuals with this risk haplotype at *DRD2* or the risk allele at *PPP1R1B* are required.

### Pathophysiological contributions of *DRD2* and *PPP1R1B* to risk for autism

The QTDT results support an association of the *DRD2* and *PPP1R1B* loci with autism (Table 4). A role for the *DRD2* gene in autism susceptibility is suggested by the fact that antipsychotic medications, which prevent dopamine D2 receptor activation, improve the core symptoms of ASDs [55]. Postsynaptic D2 receptors and presynaptic D2 autoreceptors are involved in the DAergic modulation of cognitive and emotional processes that are impaired in individuals with autism [56,57]. Thus, functional polymorphisms which affect receptor availability (e.g. altered gene expression), either postsynaptically on DAceptive neurons or presynaptically on DAergic neurons, may contribute to the impairments found in individuals with autism.

DARPP-32 mediates the downstream effects of dopamine receptor activation, and thus plays an important role in the modulation of DA-related processes which are abnormal in individuals with autism. Unlike dopamine receptors, which can be studied using systemic or local administration of ligands, DARPP-32 is found in the cytoplasm of DAceptive neurons and there are few studies which have examined its role in DA-modulated processes and behaviours. One such study by Hotte et al. [58] (2006) found that administration of D1 receptor antagonists in mice caused deficits in working memory which coincided with decreased levels of phosphorylated-DARPP-32 in the PFC. Deficiencies in working memory [59] and impairments in reversal learning [10] are found in individuals with autism. Both *Drd2* −/− mice and *Ppp1r1b* −/− mice exhibit impairments in reversal learning compared to wild-type mice [31,60].

The role of DARPP-32 in mediating the DA-related changes to neuronal excitability necessary for memory and learning was shown in a study by Calabresi et al. [61] (2000). These authors were unable to induce long-term potentiation (LTP) and long-term depression (LTD), two forms of synaptic plasticity, in the striatum of *Ppp1r1b* −/− mice. DA has a role in synaptic plasticity in both the striatum [62] and amygdala [63], subcortical structures that are important for regulating emotional behaviours [64] and social interactions [65], and are implicated in the pathophysiology of repetitive behaviours [66].

### Effects between dopamine-related genes and risk for ASD

Based on our findings in this study and our previous findings [5], single-gene analyses showed evidence for association of *DRD1DRD2* and *PPP1R1B* with autism in male-only affected sib-pair families. We performed general discriminant analysis using Statistica v9.1 to predict ASD susceptibility in affected males, and to test for evidence of gene-gene interactions of DA-related genes and ASDs. We found that *DRD2* rs1800498 genotypes and *PPP1R1B* rs1495099 genotypes significantly contributed to prediction of ASDs in our families when tested separately and together (*P* = 0.0063–0.00011), which accounted for ~7% of the variance. These results were significant following corrections for multiple comparisons (Table 5). We also found that 97% of individuals from our comparison cohort and 13% of individuals with autism were correctly classified while 72% of control individuals and 64% of affected individuals were predicted correctly using a weighted additive combination of *DRD2* and *PPP1R1B* genotypes. The inclusion of *DRD1* rs265981–rs4532–rs686 haplotypes into the analysis did not significantly change our findings (data not shown), and we found no evidence for gene-gene interactions (data not shown).

Our findings suggest that single genes individually confer risk of autism susceptibility in our families and, although there was no evidence for gene-gene interactions between DA-related genes and autism susceptibility per se, we did find evidence that *DRD2* and *PPP1R1B* together significantly contribute to ASD prediction. Thus, while both genes independently confer risk of autism susceptibility, there is a cumulative effect towards predicting whether an individual has the condition. Furthermore, based on our findings from GDA analysis we found an effect size of ~7%. While this effect size is small, it is comparable to those reported in similar studies [4]. These generally small effect sizes likely reflect the highly heterogeneous nature of ASDs.

### Parent-of-origin effects of *DRD1* and *DRD2* alleles on development

The increased transmission of the *DRD1* rs265981 C –rs4532 A –rs686 T (C-A-T) haplotype from mothers (*P* = 0.029) [5], and of the *DRD2* rs1800498 T allele from fathers (*P* = 0.023), to affected sons suggests that imprinting effects of these genes are also important risk factors for ASDs. A role for imprinting has been proposed for several brain-related disorders, including autism [67]. There is evidence of imprinting of genes from neurotransmitter pathways implicated in ASDs such as the 5-hydroxytryptamine receptor 2A (*HTR2A*) gene in the serotoninergic pathway [68].

The dopamine D1 and D2 receptors are expressed in human placentae [69,70] and fetal brains [71,72]. Placental D1 and D2 receptors are involved in DA-mediated release of opioids [73] and lactogen [74] respectively, which are required for fetal development and growth . Both dopamine D1 and D2 receptors are involved in brain development. Dopamine D2 receptors expressed in fetal brain induce neurite outgrowth and axon elongation while dopamine D1 receptors inhibit neurite outgrowth in cortical neuron differentiation [75], with the opposite effects found in striatal neuron differentiation [76]. There is no evidence for the kind of parent-of-origin-specific DNA methylation at the *DRD1* or *DRD2* loci in either human placentae or fetal brains that is suggestive of imprinting [77]. Further, a review of the ‘imprinted gene and parent-of-origin effect’ database [available at http://igc.otago.ac.nz/home.html] [78] did not yield any evidence supporting imprinting at either locus. The possibility remains, however, that imprinting of these genes may occur during a very narrow developmental period or in a specific subpopulation of brain cells, as has been demonstrated for the *Ube3a* gene in mice [79]. It should be noted that the evidence presented for parent-of-origin effects in ASDs is based on three markers in the *DRD1* gene and one marker in the *DRD2* gene and thus, more polymorphisms need to be studied to confirm our findings.

## Conclusions

Our results strongly support a role for the *DRD2* and *PPP1R1B* genes in susceptibility to autism spectrum behaviours in males from affected sib-pair families in which there are only affected males, and especially those males where there are severe impairments in social interaction (*DRD2* and *PPP1R1B*), verbal communication (*DRD2*), nonverbal communication *(PPP1R1B*) and stereotyped behaviours (*DRD2* and *PPP1R1B*). Our results also support an additive effect of the *DRD2* and *PPP1R1B* genes in predicting ASDs in our families. However, we recognize that large genome-wide association studies have not implicated these genes in autism susceptibility, but those studies did not analyse male-only affected sib-pair families separately from other families, and there is no information about the level of impairments in the core characteristics of ASDs. Further studies are needed using additional similar family cohorts and sequencing of the *DRD2* and *PPP1R1B* genes in individuals with the risk alleles to identify functional polymorphisms that could be associated with the clinical findings in order to further develop our model for an association of DA-related gene function and susceptibility to ASD behaviours.

## Competing interests

The authors declare they have no competing interests.

## Authors’ contributions

JJAH and JAH conceived and designed the study. JAH was a graduate student and this study is part of his PhD research. JAH and XL carried out the molecular analyses. MLH and AL manage the study subjects and phenotype data. ILC, RCM, CES, and MESL participated in the evaluation of the subjects. JAH, XL, and ILC performed the data analyses. JAH wrote the initial draft of the manuscript and all authors read and participated in the writing and approval of the final manuscript.
